# TNFSF15 Inhibits Blood Retinal Barrier Breakdown Induced by Diabetes

**DOI:** 10.3390/ijms17050615

**Published:** 2016-04-25

**Authors:** Feng Jiang, Qingzhong Chen, Liming Huang, Ying Wang, Zhuhong Zhang, Xiangda Meng, Yuanyuan Liu, Chunjie Mao, Fang Zheng, Jingkai Zhang, Hua Yan

**Affiliations:** Department of Ophthalmology, Tianjin Medical University General Hospital, Tianjin 300052, China; jyiyun2010@yahoo.com (F.J.); toto38359@126.com (Q.C.); lily_hlm@163.com (L.H.); wangying_1026@yahoo.com (Y.W.); zhangzhuhonghao@163.com (Z.Z.); mengxiang1989@163.com (X.M.); fangzhu1988@126.com (Y.L.); sanmaomcj@163.com (C.M.); tjmuzxf@163.com (F.Z.); zhangjingkai@163.com (J.Z.)

**Keywords:** tumor necrosis factor superfamily 15, vascular endothelial growth factor, blood retinal barrier, vascular permeability, diabetic retinopathy

## Abstract

Tumor necrosis factor superfamily 15 (TNFSF15) is an endogenous neovascularization inhibitor and an important negative regulator of vascular homeostasis. This study aimed to explore the potential role of TNFSF15 in diabetic retinopathy. Vitreous TNFSF15 and VEGF levels in proliferative diabetic retinopathy (PDR) patients were detected by ELISA. Retinal expression of TNFSF15 and the content of tight junction proteins (TJPs) in rats were detected by immunohistochemistry and Western blot, respectively. The blood retinal barrier (BRB) permeability was evaluated using Evans Blue (EB) dye. The TNFSF15/VEGF ratio was decreased in the vitreous fluid of patients with PDR relative to the controls, even though the expression levels of TNFSF15 were higher. TNFSF15 was dramatically decreased one month later after diabetes induction (*p* < 0.001), and then increased three months later and thereafter. TNFSF15 treatment significantly protected the BRB in the diabetic animals. Diabetes decreased TJPs levels in the retina, and these changes were inhibited by TNFSF15 treatment. Moreover, TNFSF15 decreased activation of VEGF both in mRNA and protein levels caused by diabetes. These results indicate that TNFSF15 is an important inhibitor in the progression of DR and suggest that the regulation of TNFSF15 shows promise for the development of diabetic retinopathy treatment strategies.

## 1. Introduction

Diabetic retinopathy (DR), the most frequent microvascular complication of diabetes, is the main cause of new blindness in working-aged adults worldwide [1,2]. In the mainland of China, the prevalence of DR is as high as 1.3% and reaches 23% in the diabetic individuals [3]. Despite its destructive consequences, the mechanisms for DR initiation and progression remain elusive. The pathogenesis of DR might be ascribed to oxidative stress [4], pro-inflammatory changes [5,6], and advanced glycation end-products [7]. There are also other important pathways involved in DR, particularly linked to VEGF, which have been discovered recently [8,9,10]. In addition to positive pathogenic regulators such as overexpression of inflammatory and angiogenic factors, loss of function of negative regulators might also contribute to disease pathogenesis [11,12]. Enhancing the endogenous negative regulators might be therapeutic for disease treatment [13,14,15,16].

Vascular endothelial growth factor (VEGF), which was identified initially as a vascular permeability factor [17], can cause an increase in the permeability of blood vessels. It is fully documented that levels of intraocular VEGF expression are increased in both diabetic rats [18,19,20] and humans [21,22,23,24], and these levels closely correlate with the BRB breakdown and retinal neovascularization. Meanwhile, anti-VEGF therapy has been shown to be beneficial in preventing retinal neovascularization and protecting the BRB [25,26]. This information suggests that VEGF plays a critical pathogenic role in the initiation and progression of DR. However, limitations to anti-VEGF therapies exist, including short duration of action, local and systemic side effects, and poor response in a notable percentage of patients [25,27,28].

The involvement of disruption of vascular homeostasis and angiogenesis in DR draws our attention to the tumor necrosis factor superfamily member 15 (TNFSF15), a newly identified cytokine [29] that is produced largely by vascular endothelial cells (ECs) of established blood vessels in normal tissue [30,31,32], but is absent or marginal in tumor vasculature in various cancers [33,34,35,36,37,38] and in wounded tissues [39]. TNFSF15 is able to induce apoptosis of proliferating ECs and prevent bone marrow-derived endothelial progenitor cells (EPCs) differentiation into ECs, which supports the view that TNFSF15 is an important negative regulator in the modulation of vascular homeostasis [36]. In addition, the role of the interaction between TNFSF15 and VEGF on the function of vascular hemostasis has been revealed preliminary. In ovarian cancer, elevated VEGF could effectively inhibit TNFSF15 production by ECs, and downregulation of TNFSF15 is a pre-requisite for tumor neovascularization [33]. In contrast, in a mouse model, TNFSF15 treatment of EPCs promotes membrane-bound VEGF receptor 1 (VEGFR1) (Flt1) (mFlt1) degradation and upregulates soluble Flt1 (sFlt1) expression in EPCs, thus giving rise to effective inhibition of VEGF-driven, EPC-supported vasculogenesis [40]. A recent study showed that TNFSF15 could upregulate tight junction proteins (TJPs) and lower the permeability of the blood brain barrier (BBB) in a mouse brain damage model by decreasing the expression of VEGF [41].

Until now, only a few studies have reported the association of TNFSF15 with ocular disease. Adenovirus-mediated expression of the C-terminal 151 residues of TNFSF15 could prevent angiogenesis in both the chick embryo chorioallantoic membrane (CAM) model and the rabbit corneal neovascularization model [42]. TNFSF15 transfection could also inhibit the length and areas of rabbit corneal neovascularization [43]. Ahmed M. *et al.* reported that TNFSF15 expression was significantly increased in SD rats and epiretinal membranes of PDR patients [44]. However, the regulation and function of TNFSF15 on the initiation and progression of DR remains elusive, and the interaction between TNFSF15 and VEGF are largely unclear as well.

In view of the critical role of TNFSF15 in modulating vascular function and the interaction of TNFSF15 with VEGF, we examined the changes in TNFSF15 level and the TNFSF15/VEGF ratio in the vitreous of proliferative diabetic retinopathy (PDR) patients as well as the retinas of streptozotocin (STZ)-induced diabetic rats, to evaluate the effect of intravitreal TNFSF15 therapy.

## 2. Results

### 2.1. TNFSF15 and VEGF Increased, but the TNFSF15/VEGF Ratio Decreased in the Vitreous Fluid of PDR Patients

**Figure 1:**
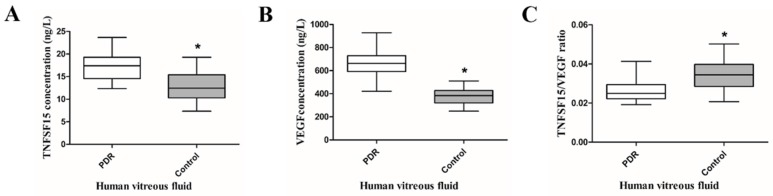
Comparison of vitreous TNFSF15 and VEGF concentrations in PDR patients and controls. * *p* = 0.000, significantly different from the PDR group (**A**) Vitreous TNFSF15 concentrations were significantly higher in PDR patients than in controls (* *p* = 0.000); (**B**) Vitreous VEGF concentrations were significantly higher in PDR patients than in controls (* *p* = 0.000); (**C**) The TNFSF15/VEGF ratio was significantly lower in PDR patients than in controls (* *p* = 0.000).

We used the ELISA assay to test the vitreous content of TNFSF15 and VEGF in patients with or without PDR. The main indications for vitrectomy in PDR patients were tractional retinal detachment (20 eyes, 57.14%) and non-clearing vitreous hemorrhage (15 eyes, 42.86%). Age was comparable between PDR patients (59.8 ± 9.15 years) and controls (62.60 ± 9.46 years) (*t* = −0.042, *p* = 0.967). Detailed data are shown in Table 1. The levels of TNFSF15 (17.17 ± 3.02 *versus* 12.86 ± 3.44 ng/L, *t* = −5.382, *p* = 0.000) and VEGF (668.50 ± 99.69 *versus* 377.71 ± 67.86 ng/L, *t* = −13.510, *p* = 0.000) in the vitreous fluid of PDR patients were significantly higher than those of controls. In contrast, the TNFSF15/VEGF ratio was dramatically decreased in PDR patients (0.0261 ± 0.0057 *versus* 0.00343 ± 0.0079, *t* = 4.859, *p* = 0.000) (Figure 1).

### 2.2. The Expression of TNFSF15 and VEGF Changed in the Retina of Diabetic Rats

**Figure 2:**
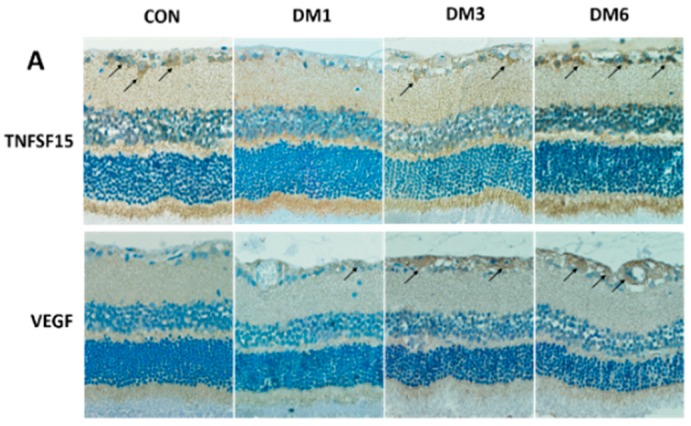

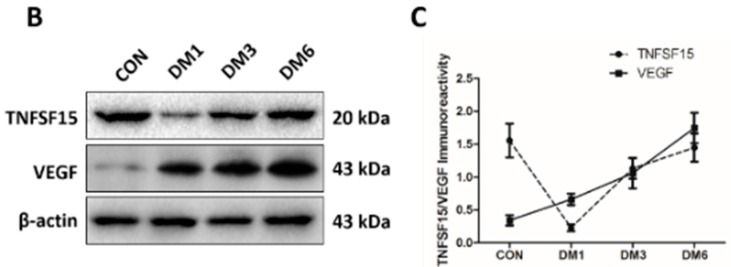
The expression of TNFSF15 and VEGF in the retina of normal rats and diabetic rats with different diabetes duration. (**A**) Typical images of TNFSF15 and VEGF immunostaining in the retina. Arrows indicate TNFSF15 (**upper** panel) or VEGF (**lower** pane) expression in the retina. Magnification, 400×; (**B**) A representative Western blot is shown; (**C**) Image of Western blot analysis is shown: TNFSF15 significantly decreased in the DM1 group compared with the CON group (*p* < 0.001), then increased gradually, with no difference between the DM6 group and the CON group (*p* = 0.351); VEGF expression increased significantly as the duration of diabetes was prolonged.

To confirm the results from patients, we established a rat DM model. The immunohistochemical results were observed by optical microscope. TNFSF15 (Figure 2A) was abundantly expressed in the retina of rats from the CON group, mostly in nerve fiber layer (NFL), ganglion cell layer (GCL), and inner nuclear layer (INL). In addition, TNFSF15 was positively expressed in the retina of rats from the DM1, DM3, and DM6 groups. Diabetes induced an obvious decrease in the TNFSF15 protein level in the retina of rats to 14.8% ± 3.4% and 72.6% ± 9.9% in the DM1 and DM3 groups compared with that of the control group, respectively (*F* = 57.738, *p* < 0.001). In contrary, there was no difference between the protein levels of the DM6 group (93.1% ± 13.8%) and the control (*p* = 0.351) (Figure 2B).

VEGF expression was elevated in the DM1, DM3, and DM6 groups. The expression of VEGF in the DM1, DM3, and DM6 groups was 1.9-, 3.1-, and 5.1-fold higher than that of the control group (*F* = 72.398, *p* < 0.001) (Figure 2A,B). These results suggest that levels of TNFSF15 were negatively correlated with those of VEGF; therefore, we supposed that TNSF15 could regulate the expression of VEGF.

### 2.3. TNFSF15 Inhibits Increased BRB Permeability Induced by Diabetes in Rats

**Figure 3:**
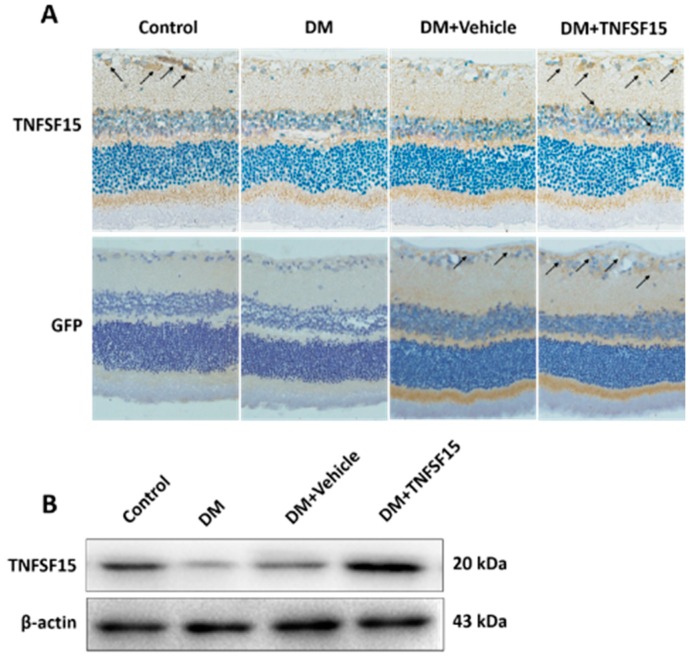
Intravitreal injection of LV-TNFSF15-GFP successfully overexpressed TNFSF15 proteins in the retina of rats. (**A**) Typical images of TNFSF15 and GFP immunostaining in the retina. Arrows indicate TNFSF15 (**upper** panel) or GFP (**lower** panel) expression in the retina. Magnification, 400×. TNFSF15 was abundantly expressed in the retina of rats treated with LV-TNFSF15-GFP, but almost deficient in rats treated with vehicle (LV-NC-GFP). GFP was abundantly expressed in the retinas of rats treated with LV-TNFSF15-GFP and LV-NC-GFP, but absent in the control and DM group; (**B**) The TNFSF15 protein level was assessed by Western blot, and a representative image is shown.

Eye tissues were dissected from the right eyes of rats from the DM+vehicle group and the DM+TNFSF15 group, and from both eyes of rats from the control group and DM group. TNFSF15 could be successfully overexpressed in the retina by injecting of LV-TNFSF15-GFP intravitreally (1 μL, 1 × 10^10^ TU/mL) 2 weeks after diabetes induction. One month after diabetes induction, the weight gain in the DM group, DM+vehicle group, and DM+TNFSF15 group was dramatically less when compared with controls, whereas the blood glucose levels were dramatically higher. The weight and the blood glucose levels in rats from the DM+TNFSF15 group were found to be similar to those of DM group (*p* = 0.551, *p* =0.692, respectively) (Table 2).These results indicate that TNFSF15 has no effect on the levels of blood glucose. When we examined GFP expression in the retina by immunohistochemistry, we found that strongly positive staining presented in all cases of animals from both DM+vehicle and DM+TNFSF15 groups, but absent on the tissue from control and DM groups (Figure 3A). Additionally, both immunohistochemical and Western blotting results showed that TNFSF15 was abundantly expressed in the retinas of rats from the DM+TNFSF15 group (Figure 3A,B).

**Figure 4:**
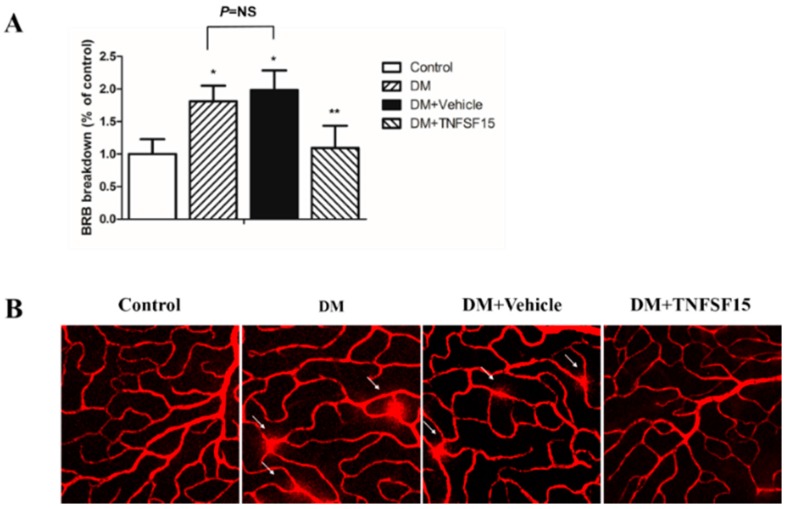
The protective effect of TNFSF15 on diabetes induced BRB permeability. (**A**) Quantitative measurement of the BRB permeability by quantification of extravasated EB. Data are presented as percentage of controls (six eyes). * *p* < 0.001, compared with the control group; ** *p* < 0.001, compared with the DM group; NS = nonsignificance; (**B**) Representative images showing EB fluorescence. In the retina of control rats, EB fluorescence is limited to the blood vessels, whereas in diabetic retina, the dye leaks out of the vessels to the retinal tissue (arrows). TNFSF15 treatment prevents the leakage of EB. Magnification 400×.

Diabetes increased BRB permeability in diabetic rats to 181.2% ± 23.9% of the control. BRB permeability was significantly decreased in diabetic rats treated with TNFSF15 (109.2% ± 34.3% of the control), but not in the ones treated with vehicle (198.4% ± 30.2% of the control) (Figure 4A). Retinal vessel leakage was also visualized with EB in retinal flat mounts. EB fluorescence was limited to the blood vessels in control retinas. However, in the retina of diabetic and vehicle-treated rats, EB leaks out of the vessels into the retinal tissue. TNFSF15 treatment prevented this effect. (Figure 4B), corroborating the results obtained from the quantitative EB assay.

### 2.4. TNFSF15 Treatment Induced the Expression of TJPs in Retinal Vessels

**Figure 5:**
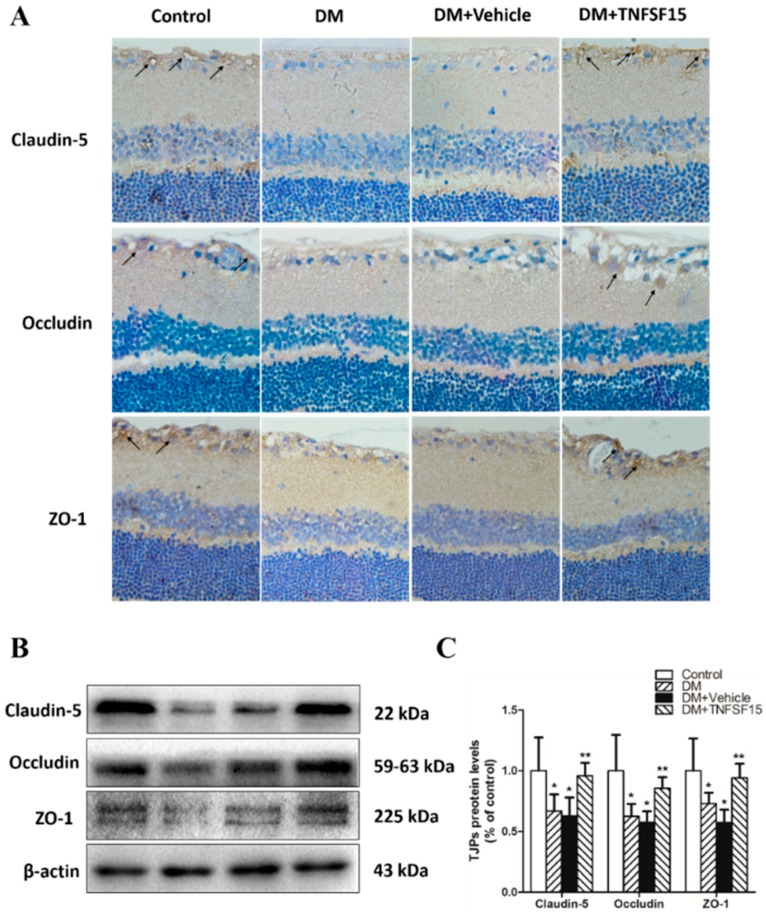
TNFSF15 prevents the decrease in claudin-5, occludin, and ZO-1 protein levels in the rat retinas induced by diabetes. (**A**) Typical images of tight junction protein immunostaining in the retina. Arrows indicate claudin-5 (**upper** panel), occluding (**middle** panel) or ZO-1 (**lower** panel) expression in the retina. Magnification 400×; (**B**) A representative Western blot is shown; (**C**) Image of a Western blot analysis is shown: the data are shown as a percentage of the control and are presented as the mean ± SD of eight eyes. ANOVA (one-way) followed by the LSD test. * *p* < 0.001, significantly different from the control group; ** *p* < 0.001, significantly different from the DM group.

To determine whether the enhanced retinal vessel leakage, observed in diabetic rats, was at least partly caused by alterations of TJPs expression, immunohistochemistry and Western blotting were used to assess the expression of claudin-5, occludin, and ZO-1, three important TJPs (Figure 5). Immunohistochemistry showed much positive staining of claudin-5, occludin, and ZO-1, mainly in GCL and INL, in rats of both control and DM+TNFSF15 groups. However, the positive staining of the three proteins was little in rats of both DM and DM+ vehicle groups (Figure 5A). Western blot was used to assess and quantify their expression further. In diabetic retinas, the expression of claudin-5, occludin, and ZO-1 decreased remarkably, to 66.7% ± 13.9%, 62.4% ± 10.2% and 72.9% ± 8.8% of the control, respectively. Treatment of diabetic rats with TNFSF15 prevented the decrease in claudin-5, occludin, and ZO-1 protein levels caused by diabetes (95.8% ± 10.6%, 85.4% ± 9.2% and 94.1% ± 11.7% of the control, respectively), indicating that TNFSF15 mediates the expression of TJPs (Figure 5B,C). These results could partly explain the effect of TNFSF15 on BRB.

### 2.5. TNFSF15 Downregulated VEGF Expression in the Retina of Diabetic Rats

**Figure 6:**
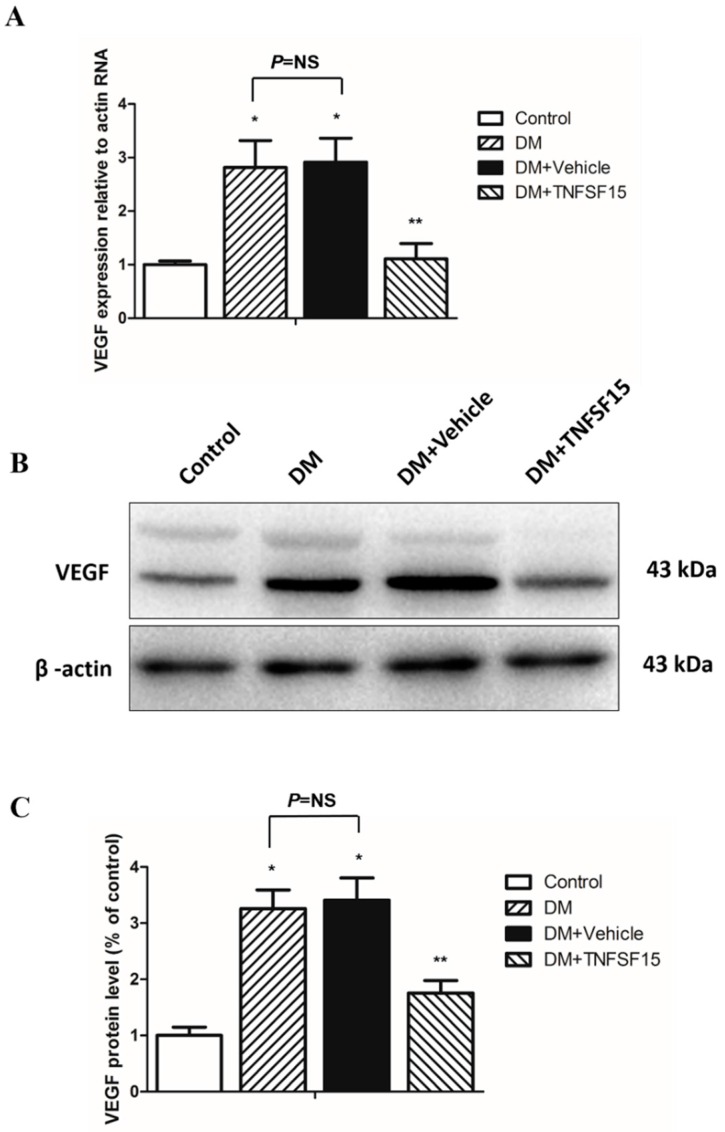
VEGF expression decreased in the retina of diabetic rats treated with TNFSF15. (**A**) RT-PCR analysis for VEGF mRNA in retinal tissue. Data are shown as the mean ± SD. ANOVA (one-way) followed by the LSD test. * *p* < 0.001, significantly different from the control group; ** *p* < 0.001, significantly different from the DM group; NS = nonsignificance; (**B**) A representative Western blot image is shown; (**C**) Image of a Western blot analysis is shown: the data are shown as a percentage of the control and are presented as the mean ± SD of eight eyes. ANOVA (one-way) followed by the LSD test. * *p* < 0.001, significantly different from the control group; ** *p* < 0.001, significantly different from the DM group; NS = nonsignificance.

The effect of TNFSF15 on the expression levels of both VEGF mRNA and protein was determined in the retina of rats with DR. The VEGF mRNA levels in the retina of the DM and DM+vehicle groups at 30 days after DM established were 281.6% ± 50.1% and 291.6% ± 44.4% of the control, respectively (*F* = 47.808, *p* < 0.001), and there was no significant difference between these two groups (*p* = 0.633). In addition, TNFSF15 treatment significantly reduced the VEGF mRNA levels (110.7% ± 28.7% of the control) (*p* < 0.001) (Figure 6A). The protein expression levels of VEGF showed the same trend; significantly increased protein levels were found in the DM and DM+vehicle groups, and TNFSF15 prevented the changes, at least in part (325.8% ± 33.5, 341.0% ± 39.6%, and 175.4% ± 22.4% of the control, respectively) (Figure 6B,C).

## 3. Discussion

In this study, we demonstrated a decreased TNFSF15/VEGF ratio in the vitreous of PDR patients, even though the expression level of TNFSF15 was increased; in diabetic animals, TNFSF15 expression was obviously decreased one month after DM induction, then increased gradually with disease progression; moreover, TNFSF15 inhibited changes in TJPs and BRB permeability induced by DR, which might be correlated with downregulation of VEGF.

It is widely accepted that vascular homeostasis is regulated by the dynamic balance of two systems: angiogenic stimulators and angiogenic inhibitors. The balance between these two systems is critical for the regulation of angiogenesis and vascular permeability [14,45]. As is well known, VEGF is the most important pathogenic factor in the initiation and progression of DR. It is fully documented that VEGF closely correlates with BRB breakdown and neovascularization. In this study, we confirmed that diabetes induced a remarkable upregulation of VEGF expression in the vitreous of PDR patients and in the retina of diabetic animals. Accordingly, the expression of TNFSF15 in diabetic rats was obviously decreased one month after DM induction, and with the prolonged duration of DM, its expression increased gradually. As DR progressed to an advanced stage, TNFSF15 expression was increased in the vitreous of PDR patients. In contrast, a significantly decreased TNFSF15/VEGF ratio was found in patients with PDR. This is inconsistent with what Abu El-Asrar *et al.* reported that TNFSF15 expression was significantly increased after four weeks of diabetes in SD rats, for its contribution to sustain the inflammatory process [44]. However, Abu El-Asrar *et al.* also revealed that TNFSF15 was overexpressed by endothelial cells of proliferating microvessels in PDR epiretinal membranes, which suggested that TNFSF15 may have a role in angiogenesis control [44]. This is quite in accord with our results. It is well known that TNFSF15 is an endogenous inhibitor of neovascularization and an important negative regulator of vascular homeostasis. Some studies showed that TNFSF15 was expressed at high levels in some vascularized tissues such as kidney, and mainly associated with established vasculature [33,38], which is consistent with the finding of our study that TNFSF15 was abundantly expressed in the retina of control rats. Deng *et al.* [33] revealed that VEGF and MCP-1 decreased TNFSF15 levels in cultured endothelial cells; this is in accord with our finding that during the early stages of DR, elevated VEGF downregulated TNFSF15 expression, resulting in disruption of the dynamic balance between the angiogenic stimulator and inhibitor systems. Therefore, vascular leakage and angiogenesis in the retina are triggered subsequently. As we know, the negative regulatory feedback loop is a critical regulatory system in the body, and as DR progresses, expression of angiogenic inhibitors (TNFSF15) was upregulated to counteract the effect of angiogenic stimulators (VEGF). Meanwhile, studies have demonstrated that the proinflammatory cytokine tumor necrosis factor α (TNF-α) is induced by VEGF in the retinal vasculature in DR [46,47], which could stimulate the expression of TNFSF15 [48]. Consequently, the level of TNFSF15 protein increased gradually with time. However, as the disease progresses, TNFSF15 is apparently not adequately potent in PDR patients to counteract the effects of angiogenic stimulators. Further investigation might be required to assess the variation of TNFSF15 expression in the eye during different stages of DR and to elucidate the signaling pathway it activates to implement its functions.

The BRB plays a key role in maintaining the homeostasis of the retinal microenvironment. Retinal vascular leakage from BRB breakdown and subsequent macular edema are the main causes of visual loss and blindness in DR. Studies of diabetic rats have indicated that BRB breakdown began as early as one week after diabetes induction [49]. Moreover, BRB breakdown is associated with changes in the expression, content, phosphorylation, and distribution of TJPs [50]. In the present study, we demonstrated that by upregulating the level of TNFSF15 in diabetic rats, the BRB permeability was significantly decreased, along with the restoration of claudin-5, occludin, and ZO-1 expression. In this study, we also demonstrated that TNFSF15 treatment downregulated VEGF expression in the retinas of diabetic rats. Thus, the elevated concentration of TNFSF15 and decreased VEGF expression might re-establish the dynamic balance between the angiogenic stimulator and inhibitor systems in the retina, thus maintaining vascular homeostasis and preventing BRB breakdown. However, the detailed mechanisms responsible for these effects remain to be elucidated as well.

## 4. Materials and Methods

### 4.1. Human Vitreous Fluid Samples

This study was approved by the medical ethics committee of Tianjin Medical University General Hospital, according to the tenets of the Declaration of Helsinki, and written informed consents were obtained from all patients. (Approval No. IRB2014-YX-009: Approval Date: 23 January 2014) Thirty-five patients (35 eyes) with PDR and 30 age-matched controls without diabetes mellitus (DM) (30 eyes) who underwent 23-gauge three-port pars plana vitrectomy (PPV) between February 2014 and August 2014 at the Department of Ophthalmology, Tianjin Medical University General Hospital were enrolled in this retrospective study. The indications for vitrectomy in individuals with PDR were tractional retinal detachment (TRD), and/or non-clearing vitreous hemorrhage (NCVH) (vitreous hemorrhage lasting more than three months before vitrectomy). All the PDR patients had type 2 DM.Patients from control group underwent vitrectomy for the treatment of idiopathic macular holes (IMH) (20 eyes) or idiopathic epiretinal membranes (ERM) (10 eyes). Patients who had previously undergone retinal photocoagulation, intravitreal anti-VEGF antibody injection or vitreoretinal surgery were excluded. Patients who had a history of ocular inflammation, high ocular pressure, rubeosis iridis, retinal vein occlusion (RVO), retinal artery occlusion (RAO), or rhegmatogenous retinal detachment (RRD) were also excluded. Vitreous fluid samples (0.3–0.6 mL) from all cases were collected by manual suction through the vitrectomy aspiration line before opening the infusion line. They were immediately centrifuged (2500× *g* for 10 min at 4 °C), and the supernatants were aliquoted and frozen at −80 °C for future use.

### 4.2. Measurement of TNFSF15 and VEGF Levels

The expression of TNFSF15 and VEGF in the human vitreous extracts was determined by enzyme-linked immunosorbent assay (ELISA), using the human TNFSF15 assay kit (R&D Systems, Minneapolis, MN, USA), and the human VEGF assay kit (R&D Systems, Minneapolis, MN, USA), respectively, following the manufacturer’s instructions. Each assay was performed in triplicate. Briefly, the vitreous fluid samples were diluted, and then 100 µL of diluted standard, blank, or sample fluid was added into the wells of a 96-well plate coated with monoclonal antibody. After two hours of incubation at 37 °C, the plate was washed, and the substrate solution was added. After the plate was incubated for 30 min, the reaction was completed by adding the stop solution, and the optical density was spectrophotometrically measured at 450 nm. A standard curve was plotted from the measurement of the standard solution. The TNFSF15 or VEGF concentration in each sample was determined from the standard curve.

### 4.3. Animals

All procedures with rats were approved by Tianjin Medical University the Laboratory Animal Care and Uses committee; and followed the Association for Research in Vision and Ophthalmology (ARVO) statement for the use of animals in ophthalmic and vision research. Male Wistar rats (Academy of Military Medical Science, Beijing, China) weighing about 200 g on arrival were used in the study. Rats were fed a commercial rat food and were allowed access to water freely in an air-conditioned room with a 12 h light/12 h dark cycle. Diabetes was induced in rats by a single intraperitoneal injection of STZ (75 mg/kg, in 10 mmoL/L citrate buffer, pH 4.5). The level of plasma glucose in each rat exceeded 300 mg/dL 48 h after injection. Rats were injected with the same volume of citrate buffer as a non-diabetic control group (CON, *n* = 8). Diabetic rats were further divided into three groups based on the time of observation: one month (DM1, *n* = 8), three months (DM3, *n* = 8), and six months (DM6, *n* = 8) groups. Plasma glucose levels were measured weekly with a self-monitoring blood glucose system (Roche, Mannheim, Germany). To keep a high survival rate, diabetic rats were subcutaneously injected (1 unit/kg body weight) with lente and regular insulin (Boots, India) daily during the experiment. Protein levels of TNFSF15 and VEGF were determined by Western blot and immunohistochemistry.

### 4.4. Intravitreal Injection

Lentivirus vector-TNFSF-15-green fluorescent protein (LV-TNFSF15-GFP) and lentivirus vector-noncontain-GFP (LV-NC-GFP) at a titer of 1 × 10^10^ TU/mL were obtained from Shanghai GeneChem Co., Ltd. (Shanghai, China). Animals were divided into the following groups: the control group (*n* = 15), DM group (*n* = 15), DM+vehicle group (intravitreally injected with LV-NC-GFP, *n* = 30) and DM+TNFSF15 group (intravitreally injected with LV-TNFSF15-GFP, *n* = 30). All the experiments were performed one month after diabetes induction. Intravitreal injection was performed two weeks after diabetes induction. Rats were anaesthetized by ketamine (64 mg/kg) and xylazine (7.2 mg/kg) intraperitoneal injection. Oxybuprocaine hydrochloride eye drops (0.4%) were applied for local anesthesia, and the pupils were dilated with tropicamide phenylephrine eye drops. Intravitreal injections (1 μL) were performed in the right eyes using a surgical microscope, a sterile Hamilton syringe (Hamilton Company, Bonaduz, Switzerland) and 33-gauge RN needles (Hamilton Company, Bonaduz, Switzerland) at 1 mm posterior to the limbus. Animals with damaged lenses or retinas by injection were excluded from the study.

### 4.5. Immunohistochemical Staining of TNFSF15, VEGF, Claudin-5, Occludin, and ZO-1

The eye tissues were fixed in 10% PBS-buffered formaldehyde, and embedded in paraffin subsequently. Serial 4 μm sections were prepared from paraffin blocks. After deparaffinization, endogenous peroxidase was quenched with 3% H_2_O_2_ in H_2_O for 5 min. Sections were incubated in 5% adult bovine serum (ABS) in PBS for one hour to minimize nonspecific adsorption. Then they were incubated overnight with anti-TNFSF15 rabbit polyclonal antibody (1:750 in 5% BSA) (Abcam, Cambridge, MA, USA), anti-GFP rabbit polyclonal antibody (1:1000 in 5% BSA) (Abcam, Cambridge, MA, USA), anti-VEGF rabbit polyclonal antibody (1:250 in 5% BSA) (Abcam, Cambridge, MA, USA), anti-claudin-5 mouse monoclonal antibody (1:100 in 5% BSA) (Invitrogen, Camarillo, CA, USA), anti-occludin rabbit polyclonal antibody (1:400 in 5% BSA) (Abcam, Cambridge, MA, USA), or anti-ZO-1 rabbit polyclonal antibody (1:400 in 5% BSA) (Invitrogen). Specific labelling was detected with biotin-conjugated goat anti-mouse IgG or goat anti-rabbit IgG and avidin-biotin peroxidase complex. Positive staining appeared brown.

### 4.6. Blood–Retinal Barrier Quantitation

The BRB breakdown in the retina was evaluated by measuring vascular permeability using an Evans Blue (EB) quantitation technique, as previously described [49], with slight modifications. Briefly, rats under deep anesthesia were injected with EB (45 mg/kg) (20 mg/mL in saline, Sigma, St. Louis, MO, USA) via the tail vein over 10 s and kept on a warm pad. Two hours after injection, 0.2 mL blood was drawn from the left ventricle, and rats were perfused via the left ventricle with about 200 mL PBS at 37 °C over a 2 min interval to clear EB from the circulation. Immediately after perfusion, the retinas were collected, thoroughly dried for 5 h, then weighed. The EB dye was extracted by incubating each retina in 120 μL of formamide (Sigma, St. Louis, MO, USA) for 18 h at 70 °C. The extract was centrifuged at 20,000× *g* for 45 min at 4 °C. The blood samples were centrifuged (12,000 rpm for 15 min) and diluted to 1/10,000th the initial concentration in formamide. The supernatant was used for spectrophotometric measurement at 620 and 740 nm. EB concentration in the extracts was calculated from a standard curve of it in formamide. The BRB breakdown was quantified by the following equation and was expressed as percentage of controls.

(1) $$\frac{\text{Retinal EB}\;\left(\mu g\right)/\text{retinal dry weight}\;\left(\text{mg}\right)}{\text{Plasma EB concentration}\;\left(\mu g/\mu L\right)\times \text{circulation time}\;\left(h\right)}$$

### 4.7. Visualization of Retinal Vessel Leakage

Retinal vascular leakage was visualized using EB. Under anesthesia, rats were injected with EB (100 mg/kg) through the tail vein, and kept on a warm pad for 120 min. Then eyes were enucleated and fixed with 4% paraformaldehyde for 2 h. The retinas were dissected flat-mounted, and examined using a confocal microscope (OLYMPUS, Tokyo, Japan) to check for EB extravasation from the retinal vessels.

### 4.8. Western Blot Analysis

Western blot was performed using standard methods. Briefly, retinas were dissected carefully from the rat eyes. Then lysates were prepared with 0.1% triton X-100 extraction buffer containing phenylmethylsulfonyl fluoride (PMSF) and dithiothreitol (DTT). The protein concentration in the tissue lysates was measured by a protein assay (Bradford Protein Assay; Bio-Rad, Hercules, CA, USA), and the protein concentrations were adjusted to allow equal total protein loading on the gels. Proteins were transferred electrophoretically to polyvinylidene fluoride (PVDF) membranes (Millipore, Billerica, MA, USA), and the membranes were blocked with 5% skim milk and then incubated with TNFSF15 (1:1000 in 5% BSA), VEGF (1:1000 in 5% BSA), claudin-5 (1:100 in 5% BSA), occludin (1:250 in 5% BSA), ZO-1 (1:250 in 5% BSA), or β-actin (1:5000 in 5% BSA) (ZSGB-BIO, Beijing, China) primary antibodies at 4 °C overnight. Then the membranes were incubated with HRP-conjugated secondary antibodies (Santa Cruz, CA, USA) at room temperature for 2 h, and visualized by the ChemiDoc™ MP System (Bio-Rad, Hercules, CA, USA), and the protein bands were quantified by Image J software for statistical analysis.

### 4.9. Quantitative Real-Time Polymerase Chain Reaction

Using an extraction reagent (TRIZOL; Invitrogen), total RNA was isolated from the retina of rats in accordance with the manufacturer’s directions for the RNA Extraction Kit (GenePharma, Hi-Tech Park, Shanghai, China). Total RNA was reverse transcribed with M-MLV reverse transcriptase (Promega, Madison, WI, USA) to generate cDNA, and the relative amounts of VEGF transcript were determined by real-time quantitative PCR. The primers used were: 5′-TTT TGC TTC CTA TTC CCC TCT T-3′ and 5′-CTC CTG CTA CCT CTT TCC TCT G-3′ for VEGF (ID:7422) and 5′-AGC CAT GTA CGT AGC CAT CC-3′ and 5′-ACC CTC ATA GAT GGG CAC AG-3′ for β-actin. The conditions of PCR were 94 °C for 30 min followed by 40 cycles at 95 °C for 20 s; 57 °C for 20 s; and 72 °C for 20 s. Measurements were performed on six samples from one group three times independently.

### 4.10. Statistics

All data are presented as the means ± SD. Data were analyzed by the independent sample student’s *t*-test for two comparisons and one-way ANOVA followed by the LSD test for multiple comparisons, as indicated in the figure legends. Differences were considered statistically significant when *p* < 0.05.

## 5. Conclusions

Our data demonstrated that the actions of TNFSF15 and VEGF might demonstrate an important mechanism for maintaining vascular homeostasis. Intravitreal administration of TNFSF15 significantly inhibits BRB breakdown in STZ-diabetic rats. These results confirm the clinical potential of TNFSF15 for the treatment of diabetes-induced retinal vasculopathy.

## Figures and Tables

**Table 1:** Patient characteristics.

Variable	PDR (*n* = 35)	Control (*n* = 30)	*p* Value
Age (years)	59.8 ± 9.15	62.60 ± 9.46	0.230
Gender/male (%)	16 (45.7)	14 (46.7)	0.939
Duration of diabetes (years)	14.74 ± 8.07	-	-
HbA1c (%)	8.06 ± 1.76	4.60 ± 0.69	0.000
Glucose (mmol/L)	7.29 ± 1.76	4.97 ± 0.94	0.000

PDR = proliferative diabetic retinopathy; HbA1c = glycosylated hemoglobin.

**Table 2:** Weight and blood glucose levels of rats from different groups.

	Weight (g)	Glycaemia (mg/dL)	*N*
Control	293.9 ± 12.9	82.08 ± 6.3	15
DM	249.9 ± 13.4	456.48 ± 57.6	15
DM+vehicle	250.4 ± 12.8	461.34 ± 52.74	30
DM+TNFSF15	251.8 ± 12.9	461.52 ± 55.62	30
*F* value	75.583	376.721	-
*p* value	0.000	0.000	-

DM = diabetes mellitus.

## Abbreviations

TNFSF15	tumor necrosis factor superfamily 15
VEGF	vascular endothelial growth factor
PDR	proliferative diabetic retinopathy
TJPs	tight junction proteins
BRB	blood retinal barrier
EB	Evans Blue
DR	diabetic retinopathy
ECs	endothelial cells
EPC	endothelial progenitor cell
mFlt1	membrane-bound VEGF receptor 1
sFlt1	soluble Flt1
BBB	blood brain barrier
CAM	chorioallantoic membrane
STZ	streptozotocin
NFL	nerve fibre layer
GCL	ganglion cell layer
INL	inner nuclear layer
MCP-1	monocyte chemoattractant protein-1
TNF	tumor necrosis factor
DM	diabetes mellitus
PPV	pars plana vitrectomy
TRD	tractional retinal detachment
NCVH	non-clearing vitreous haemorrhage
IMH	idiopathic macular holes
ERM	idiopathic epiretinal membranes
RVO	retinal vein occlusion
RAO	retinal artery occlusion
RRD	rhegmatogenous retinal detachment
ELISA	enzyme-linked immunosorbent assay
ARVO	the Association for Research in Vision and Ophthalmology
LV-TNFSF15-GFP	lentivirus vector-TNFSF-15-green fluorescent protein
LV-NC-GFP	lentivirus vector-noncontain-GFP
PMSF	phenylmethylsulfonyl fluoride
DTT	dithiothreitol
PVDF	polyvinylidene fluoride

## References

[1] Klein B.E. (2007). Overview of epidemiologic studies of diabetic retinopathy. Ophthalmic Epidemiol..

[2] Yau J.W., Rogers S.L., Kawasaki R., Lamoureux E.L., Kowalski J.W., Bek T., Chen S.J., Dekker J.M., Fletcher A., Grauslund J. (2012). Global prevalence and major risk factors of diabetic retinopathy. Diabetes Care.

[3] Liu L., Wu X., Liu L., Geng J., Yuan Z., Shan Z., Chen L. (2012). Prevalence of diabetic retinopathy in mainland China: A meta-analysis. PLoS ONE.

[4] Kowluru R.A., Chan P.S. (2007). Oxidative stress and diabetic retinopathy. Exp. Diabetes Res..

[5] Tang J., Kern T.S. (2011). Inflammation in diabetic retinopathy. Prog. Retin. Eye Res..

[6] Zhang W., Liu H., Rojas M., Caldwell R.W., Caldwell R.B. (2011). Anti-inflammatory therapy for diabetic retinopathy. Immunotherapy.

[7] Zong H., Ward M., Stitt A.W. (2011). AGEs, RAGE, and diabetic retinopathy. Curr. Diabetes Rep..

[8] Amadio M., Bucolo C., Leggio G.M., Drago F., Govoni S., Pascale A. (2010). The PKCbeta/HuR/VEGF pathway in diabetic retinopathy. Biochem. Pharmacol..

[9] Lupo G., Motta C., Giurdanella G., Anfuso C.D., Alberghina M., Drago F., Salomone S., Bucolo C. (2013). Role of phospholipases A2 in diabetic retinopathy: *In vitro* and *in vivo* studies. Biochem. Pharmacol..

[10] Giurdanella G., Anfuso C.D., Olivieri M., Lupo G., Caporarello N., Eandi C.M., Drago F., Bucolo C., Salomone S. (2015). Aflibercept, bevacizumab and ranibizumab prevent glucose-induced damage in human retinal pericytes *in vitro*, through a PLA2/COX-2/VEGF-A pathway. Biochem. Pharmacol..

[11] Zhang C., Wang H., Nie J., Wang F. (2014). Protective factors in diabetic retinopathy: Focus on blood-retinal barrier. Discov. Med..

[12] Mohan N., Monickaraj F., Balasubramanyam M., Rema M., Mohan V. (2012). Imbalanced levels of angiogenic and angiostatic factors in vitreous, plasma and postmortem retinal tissue of patients with proliferative diabetic retinopathy. J. Diabetes Complicat..

[13] Wang J.J., Zhang S.X., Lu K., Chen Y., Mott R., Sato S., Ma J.X. (2005). Decreased expression of pigment epithelium-derived factor is involved in the pathogenesis of diabetic nephropathy. Diabetes.

[14] Zhang S.X., Wang J.J., Gao G., Parke K., Ma J.X. (2006). Pigment epithelium-derived factor downregulates vascular endothelial growth factor (VEGF) expression and inhibits VEGF-VEGF receptor 2 binding in diabetic retinopathy. J. Mol. Endocrinol..

[15] Rask-Madsen C., King G.L. (2010). Kidney complications: Factors that protect the diabetic vasculature. Nat. Med..

[16] Jeong I.K., King G.L. (2011). New perspectives on diabetic vascular complications: The loss of endogenous protective factors induced by hyperglycemia. Diabetes Metab. J..

[17] Weis S.M., Cheresh D.A. (2005). Pathophysiological consequences of VEGF-induced vascular permeability. Nature.

[18] Qaum T., Xu Q., Joussen A.M., Clemens M.W., Qin W., Miyamoto K., Hassessian H., Wiegand S.J., Rudge J., Yancopoulos G.D. (2001). VEGF-initiated blood-retinal barrier breakdown in early diabetes. Investig Ophthalmol. Vis. Sci..

[19] Murata T., Nakagawa K., Khalil A., Ishibashi T., Inomata H., Sueishi K. (1996). The relation between expression of vascular endothelial growth factor and breakdown of the blood-retinal barrier in diabetic rat retinas. Lab. Investig..

[20] Gilbert R.E., Vranes D., Berka J.L., Kelly D.J., Cox A., Wu L.L., Stacker S.A., Cooper M.E. (1998). Vascular endothelial growth factor and its receptors in control and diabetic rat eyes. Lab. Investig..

[21] Wang J., Chen S., Jiang F., You C., Mao C., Yu J., Han J., Zhang Z., Yan H. (2014). Vitreous and plasma VEGF levels as predictive factors in the progression of proliferative diabetic retinopathy after vitrectomy. PLoS ONE.

[22] Matsuyama K., Ogata N., Jo N., Shima C., Matsuoka M., Matsumura M. (2009). Levels of vascular endothelial growth factor and pigment epithelium-derived factor in eyes before and after intravitreal injection of bevacizumab. Jpn. J. Ophthalmol..

[23] Costagliola C., Daniele A., dell'Omo R., Romano M.R., Aceto F., Agnifili L., Semeraro F., Porcellini A. (2013). Aqueous humor levels of vascular endothelial growth factor and adiponectin in patients with type 2 diabetes before and after intravitreal bevacizumab injection. Exp. Eye Res..

[24] Sawada O., Kawamura H., Kakinoki M., Sawada T., Ohji M. (2007). Vascular endothelial growth factor in aqueous humor before and after intravitreal injection of bevacizumab in eyes with diabetic retinopathy. Arch. Ophthalmol..

[25] Simo R., Sundstrom J.M., Antonetti D.A. (2014). Ocular Anti-VEGF therapy for diabetic retinopathy: The role of VEGF in the pathogenesis of diabetic retinopathy. Diabetes Care.

[26] Ip M.S., Domalpally A., Hopkins J.J., Wong P., Ehrlich J.S. (2012). Long-term effects of ranibizumab on diabetic retinopathy severity and progression. Arch. Ophthalmol..

[27] Agard E., Elchehab H., Ract-Madoux G., Russo A., Lagenaite C., Dot C. (2015). Repeated intravitreal anti-vascular endothelial growth factor injections can induce iatrogenic ocular hypertension, especially in patients with open-angle glaucoma. Can. J. Ophthalmol..

[28] Cheungpasitporn W., Chebib F.T., Cornell L.D., Brodin M.L., Nasr S.H., Schinstock C.A., Stegall M.D., Amer H. (2015). Intravitreal Antivascular Endothelial Growth Factor Therapy May Induce Proteinuria and Antibody Mediated Injury in Renal Allografts. Transplantation.

[29] Tan K.B., Harrop J., Reddy M., Young P., Terrett J., Emery J., Moore G., Truneh A. (1997). Characterization of a novel TNF-like ligand and recently described TNF ligand and TNF receptor superfamily genes and their constitutive and inducible expression in hematopoietic and non-hematopoietic cells. Gene.

[30] Chew L.J., Pan H., Yu J., Tian S., Huang W.Q., Zhang J.Y., Pang S., Li L.Y. (2002). A novel secreted splice variant of vascular endothelial cell growth inhibitor. FASEB J..

[31] Zhai Y., Ni J., Jiang G.W., Lu J., Xing L., Lincoln C., Carter K.C., Janat F., Kozak D., Xu S. (1999). VEGI, a novel cytokine of the tumor necrosis factor family, is an angiogenesis inhibitor that suppresses the growth of colon carcinomas *in vivo*. FASEB J..

[32] Zhai Y., Yu J., Iruela-Arispe L., Huang W.Q., Wang Z., Hayes A.J., Lu J., Jiang G., Rojas L., Lippman M.E. (1999). Inhibition of angiogenesis and breast cancer xenograft tumor growth by VEGI, a novel cytokine of the TNF superfamily. Int. J. Cancer.

[33] Deng W., Gu X., Lu Y., Gu C., Zheng Y., Zhang Z., Chen L., Yao Z., Li L.Y. (2012). Down-modulation of TNFSF15 in ovarian cancer by VEGF and MCP-1 is a pre-requisite for tumor neovascularization. Angiogenesis.

[34] Parr C., Gan C.H., Watkins G., Jiang W.G. (2006). Reduced vascular endothelial growth inhibitor (VEGI) expression is associated with poor prognosis in breast cancer patients. Angiogenesis.

[35] Zhou J., Yang Z., Tsuji T., Gong J., Xie J., Chen C., Li W., Amar S., Luo Z. (2011). LITAF and TNFSF15, two downstream targets of AMPK, exert inhibitory effects on tumor growth. Oncogene.

[36] Zhang Z., Li L.Y. (2012). TNFSF15 Modulates Neovascularization and Inflammation. Cancer Microenviron..

[37] Zhang N., Sanders A.J., Ye L., Kynaston H.G., Jiang W.G. (2010). Expression of vascular endothelial growth inhibitor (VEGI) in human urothelial cancer of the bladder and its effects on the adhesion and migration of bladder cancer cells *in vitro*. Anticancer Res..

[38] Zhang N., Sanders A.J., Ye L., Jiang W.G. (2009). Vascular endothelial growth inhibitor in human cancer (Review). Int. J. Mol. Med..

[39] Conway K.P., Price P., Harding K.G., Jiang W.G. (2007). The role of vascular endothelial growth inhibitor in wound healing. Int. Wound J..

[40] Qi J.W., Qin T.T., Xu L.X., Zhang K., Yang G.L., Li J., Xiao H.Y., Zhang Z.S., Li L.Y. (2013). TNFSF15 inhibits vasculogenesis by regulating relative levels of membrane-bound and soluble isoforms of VEGF receptor 1. Proc. Natl. Acad. Sci. USA.

[41] Gao W., Zhao Z., Yu G., Zhou Z., Zhou Y., Hu T., Jiang R., Zhang J. (2015). VEGI attenuates the inflammatory injury and disruption of blood-brain barrier partly by suppressing the TLR4/NF-kappaB signaling pathway in experimental traumatic brain injury. Brain Res..

[42] Pan X., Wang Y., Zhang M., Pan W., Qi Z.T., Cao G.W. (2004). Effects of endostatin-vascular endothelial growth inhibitor chimeric recombinant adenoviruses on antiangiogenesis. World J. Gastroenterol..

[43] Wang H., Wang B. (2010). Inhibition of corneal neovascularization by vascular endothelia growth inhibitor gene. Int. J. Ophthalmol..

[44] Abu El-Asrar A.M., De Hertogh G., Nawaz M.I., Siddiquei M.M., van den Eynde K., Mohammad G., Opdenakker G., Geboes K. (2015). The Tumor Necrosis Factor Superfamily Members TWEAK, TNFSF15 and Fibroblast Growth Factor-Inducible Protein 14 Are Upregulated in Proliferative Diabetic Retinopathy. Ophthalmic Res..

[45] Bussolino F., Mantovani A., Persico G. (1997). Molecular mechanisms of blood vessel formation. Trends Biochem. Sci..

[46] Huang H., Gandhi J.K., Zhong X., Wei Y., Gong J., Duh E.J., Vinores S.A. (2011). TNFalpha is required for late BRB breakdown in diabetic retinopathy, and its inhibition prevents leukostasis and protects vessels and neurons from apoptosis. Investig. Ophthalmol. Vis. Sci..

[47] Vinores S.A., Xiao W.H., Shen J., Campochiaro P.A. (2007). TNF-α is critical for ischemia-induced leukostasis, but not retinal neovascularization nor VEGF-induced leakage. J. Neuroimmunol..

[48] Xu L.X., Grimaldo S., Qi J.W., Yang G.L., Qin T.T., Xiao H.Y., Xiang R., Xiao Z., Li L.Y., Zhang Z.S. (2014). Death receptor 3 mediates TNFSF15- and TNFalpha-induced endothelial cell apoptosis. Int. J. Biochem. Cell Biol..

[49] Xu Q., Qaum T., Adamis A.P. (2001). Sensitive blood-retinal barrier breakdown quantitation using Evans blue. Investig. Ophthalmol. Vis. Sci..

[50] Klaassen I., Van Noorden C.J., Schlingemann R.O. (2013). Molecular basis of the inner blood-retinal barrier and its breakdown in diabetic macular edema and other pathological conditions. Prog. Retin. Eye Res..

